# A microarray-based pathogen chip for simultaneous molecular detection of transfusion–transmitted infectious agents

**DOI:** 10.1186/s12967-019-1905-4

**Published:** 2019-05-14

**Authors:** Valeria De Giorgi, Huizhi Zhou, Harvey J. Alter, Robert D. Allison

**Affiliations:** Infectious Diseases Section, Department of Transfusion Medicine, National Institutes of Health Clinical Center, Building 10, Room 1C711, 10 Center Drive, Bethesda, MD 20892 USA

**Keywords:** Blood donor screening, Blood safety, Transfusion–transmitted infections, RNA virus, Microarray

## Abstract

**Background:**

New and emerging transfusion–transmitted infections remain a threat to the blood supply. Blood donors are currently screened for less than half of known agents, primarily by individual tests. A screening platform that could simultaneously detect all known transfusion–transmitted pathogens and allow rapid addition of new targets would significantly increase blood safety and could improve the response to new agents. We describe the early stage development and validation of a microarray-based platform (pathogen chip) for simultaneous molecular detection of transfusion–transmitted RNA viruses.

**Methods:**

Sixteen RNA viruses that pose a significant risk for transfusion–transmission were selected for inclusion on the pathogen chip. Viruses were targeted for detection by 1769 oligonucleotide probes selected by Agilent eArray software. Differentially concentrated positive plasma samples were used to evaluate performance and limits of detection in the context of individual pathogens or combinations to simulate coinfection. RNA-viruses detection and concentration were validated by RT-qPCR.

**Results:**

Hepatitis A, B and C, Chikungunya, dengue 1–4, HIV 1–2, HTLV I–II, West Nile and Zika viruses were all correctly identified by the pathogen chip within the range of 10^5^ to 10^2^ copies/mL; hepatitis E virus from 10^5^ to 10^4^. In mixtures of 3–8 different viruses, all were correctly identified between 10^5^ and 10^3^ copies/mL.

**Conclusions:**

This microarray-based multi-pathogen screening platform accurately and reproducibly detected individual and mixed RNA viruses in one test from single samples with limits of detection as low as 10^2^ copies mL.

**Electronic supplementary material:**

The online version of this article (10.1186/s12967-019-1905-4) contains supplementary material, which is available to authorized users.

## Background

Each year over 112 million blood donations are collected globally and nearly 21 million blood components are transfused in the U.S. [1]. Though screening of these blood units using serologic and nucleic acid testing (NAT) has greatly reduced the risk of some transfusion–transmitted infections, the vast majority of bloodborne agents are not screened [2–4]. The U.S. Food and Drug Administration (FDA) licensed methods for infectious disease screening of donor blood include (1) NAT for Hepatitis B virus (HBV), Hepatitis C virus (HCV), HIV 1 and 2, Babesia, West Nile virus (WNV) and Zika virus (ZIKV); and (2) immunoassays for HBV, HCV, HIV-1, 2, cytomegalovirus (CMV), human T cell lymphotropic virus I and II (HTLV), *Treponema* *pallidum* (syphilis) and *Trypanosoma cruzi* (Chagas). HTLV, syphilis and Chagas antibody testing fail to detect these pathogens during early infection and Chagas is screened only once on samples from first-time blood donors [5].

The American Association of Blood Banks (AABB) Transfusion–Transmitted Diseases Committee produced a list of over 30 pathogens of concern for transmission via blood that included bacteria, parasites, prions and viruses [6]. Only prions cannot be detected by currently available technology. Nearly all the other agents require individual qPCR or serologic testing and it is logistically impractical and cost prohibitive to test all known and potential agents individually [7–9].

Multiplex PCR-based devices for testing blood-borne pathogens are limited. Two FDA-approved blood donor screening assays (Cobas Taqscreen MPX Test, Roche Molecular Systems, Inc. and the Procleix Ultrio Plus,Gen-Probe, Inc.) use PCR or transcription-mediated amplification methodology for multiplex detection of HBV, HCV, and HIV 1–2 [5]. A multiplex assay capable of detecting all known pathogens of concern in a single small blood sample with high sensitivity and specificity could significantly increase the safety of the blood supply. Further, to counter emerging pathogens, the platform should be adaptable for rapid addition and validation of probes to detect new agents. Microarray-based technology offers the advantage of multiplex detection in a miniaturized format with high adaptability. The presence of multiple probes per target represent an advantage in comparison to traditional NAT or EIA assays since the pathogen can be detected even if mutations block the effectiveness of some probes [10]. The flexibility and high-throughput capability of microarrays hold great potential for pathogen detection and identification, but historically have limitations in detecting agents present at low copy numbers. [11–13]. To optimize performance and improve detection limits, we implemented two strategies: (1) a platform design that simultaneously detects and distinguishes multiple pathogens and closely related viral strains; and (2) an innovative combination of amplification and labeling protocols to detect multiple targets present at low levels in a single sample.

In this proof of concept study, we evaluated the performance of a customized microarray-based pathogen chip for simultaneous detection of 16 RNA viruses in human plasma samples that is designed to have the flexibility to expand to detect emerging agents in a relatively short time frame. A parallel, and ultimately integrated, chip to detect DNA viruses, bacteria and parasites is in development. Platform performance was evaluated using positive plasma donor samples and pathogen-spiked plasma.

## Methods

### Selection of transfusion–transmitted RNA viruses and oligonucleotide probes

Sequences of 16 RNA viruses of concern for transmission to blood recipients (released by AABB Transfusion–Transmitted Diseases Committee [6]) were downloaded from Gen-bank at NCBI (https://www.ncbi.nlm.nih.gov/genbank).

The complete genome for each RNA virus was uploaded in FASTA format using Agilent eArray software (https://earray.chem.agilent.com/earray/Agilent Technologies Inc., Santa Clara, CA). Design settings were chosen to select 60-mer sense probes with 3′ bias from each viral gene, according to the base composition methodology, which considers fusion temperature, GC% and cross-hybridization potential for probes. To get the best quality level probes for viral genome detection we selected “best probe” (BP). The probes were checked for vector and low complexity masking. Entire viral genome sequences were covered to the extent possible with all available Agilent-designed probes. The microarray was supplemented with additional predesigned GE (gene expression) array probes for 906 genes from the human genome (replicated 10 times), ERCC probes (replicated 45 times) and probes covering plant virus sequences (negative control).

Oligonucleotide probes were synthesized in situ from 3′-end base by base with Agilent SurePrint inkjet technology according to the manufacturer’s protocol [14]. The microarrays were manufactured with 60-mer oligonucleotides synthesized in 15,000 features on eight replicate arrays per slide.

### Sample collection and processing

Specimens positive for CHIKV, DENV1–4, HIV1–2, WNV and ZIKV were prepared, validated and kindly supplied by the FDA Center for Biologics Evaluation and Research (CBER) [15].

HCV genotypes 1a, 2a, and 3, and HEV RNA-positive plasma were purchased from Sera Care (Sera Care, Milford, MA). All positive specimens were diluted in negative plasma (Basematrix diluent, Sera Care) to create a range of concentrations. HAV RNA was obtained from Dr. Patrizia Farci, (National Institutes of Health, Bethesda, MD). HTLV types I and II NATtrol (Nucleic Acid Testing Control) were purchased from ZeptoMetrix (ZeptoMetrix, Buffalo, NY) Additional file 1: Table S1.

Nucleic acids from positive plasma and from NATtrol were extracted using the Dynabeads™ SILANE Viral NA Kit (ThermoFisher Scientific, Waltham, MA) according to the manufacturer’s protocol.

cDNA from random-primed, reverse-transcribed total RNA was performed with the Ovation Pico WTA System (NuGEN, San Carlos, CA) using the manufacturer’s recommended protocols and input amounts. For this study, the Agilent SureTag Labeling Kit was used for generating Cy3 labeled cDNA targets. Labeled cDNA was purified with SureTag Kit spin columns and specific activities (degree of labeling) were calculated for use in hybridization reactions. A master mix containing 10× blocking agent and 2× GE hybridization buffer HI-RPM, was added to 3–5 μg of labeled cDNA, denatured, and hybridized to arrays under 8-chamber gasket slides at 65 °C with 20-rpm rotation for 24 h in an Agilent hybridization oven. Arrays were processed using wash procedure A and scanned on an Agilent SureScan G4900DA microarray scanner using 5-µm resolution.

### Novel workflow to enhance amplification

One of the challenges impacting the sensitivity of microarray-based multi-pathogen nucleic acid detection in blood specimens is the relatively small concentration of target nucleic acids compared to a high background concentration of human DNA. We designed a novel workflow combining two different applications that had not been previously combined, to address this challenge. Typically the Agilent amplification WT kit (Oligo dT) is used to amplify total RNA, with a minimum nucleic acid requirement of 25 ng, and produces a cRNA final product that is labelled with Cy3 fluorophore. We modified the workflow using a method that generates amplified cDNA from as little as 500 picograms of target viral RNA. One single-primer isothermal amplification using Nugen Ribo-SPIA technology was combined with the Agilent Genomic DNA Enzymatic Labeling Kit for generating Cy3 labeled cDNA. This application was not previously developed for single color RNA probes.

### Data analyses

After scanning, microarray images were analyzed using Agilent Feature Extraction software (Agilent Technologies, Inc., Santa Clara, CA) with default protocols and settings. Average pixel intensity and subtraction of local background for each feature was calculated. Images were manually examined to note any arrays affected by high background, scratches, or other technical artifacts. Probe sets associated with low signal intensity or bad quality features were considered unreliable and excluded from the analysis. Feature intensities for Cy3 channels were imported into the Partek Genomics Suite (Partek Inc., St. Louis, MO).

First, microarray analysis was performed by ranking the highest signal intensity probes by the mean of the set of probes defining each pathogen on the platform. Next, an experimental threshold was defined as a log ratio of signal intensity mean for the set of probes defining each pathogen and the mean of the Agilent control probes set. The threshold was applied to all the arrays tested to define the final parameters for test validation.

Quality of signals generated by probes for each species was assessed according to two experimental criteria: (1) define a threshold able to distinguish a true signal; (2) define true positives when only 50% of probes generated a signal above the set threshold. These two levels of data analysis were needed to detect positive probes in the simultaneous presence of multiple pathogens at varying concentrations. The threshold was defined as the log ratio between the signal intensity mean for each pathogen specific probe set and the mean of the Agilent control group probe set. After comparing the results of the same set of probes across different arrays and selecting the probes showing an inter-array reproducibility, an experimental threshold value was defined as follows: log ratio < 1 negative; log ratio ≥ 1.0 to ≤ 1.5 borderline; log ratio > 1.5 positive.

**Fig. 1:**
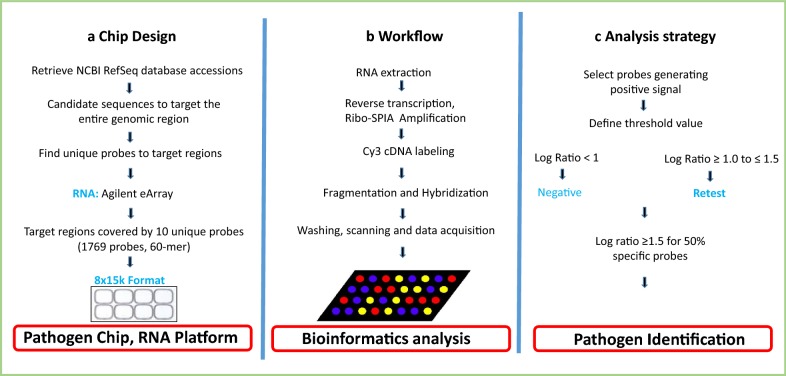
Pathogen chip design, plasma samples preparation workflow and analysis strategy. **a** Sequence accessions for all RNA viruses were retrieved from the NCBI genome sequence databases and candidate as sequences to target. Regions of target sequence unique to the accession were used to select multiple 60-mer probes for microarray synthesis. **b** A novel workflow design combining two different applications generating cDNA instead of amplified RNA, followed by Cy3 labelling and hybridization based on a DNA application. **c** Experimental criteria to assess quality of signals generated by probes for each species

Data analysis at the individual probe level was also performed to assess if the tested samples were false positives. The test was considered valid only when at least 50% of specific probes had log ratio > 1.5 (Fig. 1c).

### RT-qPCR Validation

CHIKV, DENV 1–4 and ZIKV positive specimens were quantified using the Altona RealStar RT-qPCR kit (Altona Diagnostic GmbH., Hamburg, Germany) according to the manufacturer’s instructions. The positive and internal controls were provided by the manufacturer. Serial dilutions of CHIKV (ATCC VR-3246SD), DENV (ATCC VR-3231SD) and ZIKV (ATCC VR-1843DQ) quantitative genomic RNA (specification range: 1 × 10^5^–1 × 10^6^ copies/µL) obtained from the American Type Culture Collection (ATCC, Manassas, VA) were prepared to generate a standard curve for copy number quantification.

HAV (target/5′ NCR), HCV (5′UTR), HEV (ORF2), HIV-1 (target/POL), HIV-2 (target/POL), HTLVI (target/POL), HTLVII (target/POL), and WNV (5′UTR) positive specimens were quantified using the Primer Design Genesig kit (Primerdesign Ltd, United Kingdom) according to the manufacturer’s protocol (OneStep RT-qPCR protocol). Each kit contained a positive control template for the PCR set up and for copy number determination (generated serial dilutions for the standard curve).

The RT-qPCR assays were performed on a ViiA7 Applied Biosystems real-time PCR system (Thermo Fisher Scientific Inc., Waltham, MA). Each sample was tested in duplicate and the mean Cq value was calculated.

## Results

The pathogen chip design strategy was to cover all high priority bloodborne RNA viruses (retroviruses, and both positive- and negative-strand RNA viruses) using multiple probes to independent target sites in the genome of each species. In total, 1769 unique viral oligonucleotides derived from 16 distinct viral genomes were selected that allowed discrimination of pathogens at the level of species, subtypes and genotypes (Table 1). The final microarray design was supplemented with negative controls, including predesigned GE array probes for 906 genes from the human genome, 84 ERCC probes and 120 probes specific for plant viruses (Table 1). The eArray quality scoring evaluation (BC quality scores: BC1, high; BC3, low) revealed that nearly 90% of the selected probes had a high quality score (87% BC1, 13% BC2, 0.1% BC3).

**Table 1:** Probes distribution on pathogen chip

Probe group type	Number of targets	Number of probes	Purpose
All spot	1010	14,716	RNA pathogens coverage and internal controls
Pathogen specific (not replicated)	16	1769	Probes intensity analysis of pathogen specific genes
Internal control (replicated 10 times)	902	902	Agilent requirement for probes normalization
ERCC probes (replicated 45 times)	84	84	Determination of intra-probe variance
Negative control (not replicated)	3	120	Determination of probes cross reactivity

The design included multiple gene targets for each pathogen genome in order to select the best probes for the final platform design. Probes selected in the final design generated a more intense signal and produced higher percentage coverage of the specific genome across the different experiments (Additional file 2: Figure S1a, b).

### Amplification enhancement using novel workflow

Our novel workflow design combining two different applications produced up to 300% of the amplified product compared to the standard methodology (Fig. 1b and Additional file 3: Figure S2a). Nearly all samples were detected on the platform and all probes generated a strong signal specific for each positive plasma specimen analyzed. No specific signal was produced by negative control plasma (Additional file 3: Figure S2b). Random nonspecific intensity signal was produced in only a few arrays. This indicated that the generation of cDNA instead of amplified RNA followed by Cy3 labelling and hybridization based on a DNA application was successful (Additional file 3: Figure S2c).

### Quality of probe signals

For nearly all borderline results, only 20–25% of the specific probes showed mean intensity in the correct range, so the test result was considered negative. For positive results (log ratio > 1.5) more than 50% of the specific probe sets were in the correct range. One example was an HCV 1a positive plasma samples test that was detected by 110 out of 110 probes at a concentration of 10^5^ copies/mL, 90 out of 110 probes at a concentration of 10^4^ copies/mL and 70 out of 110 probes at a concentration of 10^3^ copies/mL. At 10^2^ copies/mL, on average more than 50% of the probes generated a fluorescence signal above the set threshold.

Data from more than 168 tested samples (one or multiple targets, randomly mixed, per array) showed consistent results. The mean of the probes specific for any positive plasma sample was always at least tenfold higher than the mean of internal control probes (background), showing a wide range of intensity within one probe population. As shown in Tables 2 and 3, the Log Ratio was above 1.5 for all the pathogens tested at a concentration of 10^2^ copies/mL and there were no cross reactions with other probes across the platform.

**Table 2:** Test results based on log ratio

	CK	DEN1	DEN2	DEN3	DEN4	HAV	HCV1a	HCV2a	HCV3	HEV	HIV-1	HIV-2	HTLVI	HTLVII	WNV	ZKV	NC
CK	*2.42*	− 1.15	− 1.22	− 0.04	− 0.28	− 0.50	− 0.24	− 0.35	− 0.56	− 0.02	− 0.99	− 0.68	− 0.42	− 0.96	− 0.97	− 0.20	− 0.96
DEN1	− 0.12	*1.60*	− 0.33	0.09	0.14	− 0.08	− 0.20	− 0.31	− 0.52	0.46	− 0.76	− 0.55	− 0.28	− 0.76	− 0.53	0.00	− 0.86
DEN2	− 0.24	− 0.82	*1.84*	− 0.30	− 0.25	− 0.24	− 0.17	− 0.28	− 0.49	− 0.11	− 1.13	− 0.89	− 0.52	− 0.84	− 1.00	− 0.28	− 1.01
DEN3	0.03	− 0.19	− 0.02	*1.62*	− 0.24	− 0.28	− 0.24	− 0.34	− 0.55	0.01	− 0.95	− 0.81	− 0.56	− 0.91	− 0.22	0.05	− 0.96
DEN4	0.07	− 0.31	− 0.64	0.18	*1.80*	− 0.64	− 0.17	− 0.28	− 0.49	− 0.20	− 1.02	− 0.80	− 0.66	− 1.05	− 0.30	− 0.36	− 1.07
HAV	− 0.06	− 0.97	− 1.24	− 0.25	− 0.34	*2.97*	− 0.21	− 0.31	− 0.52	0.35	− 0.78	− 0.47	− 0.35	− 0.71	− 1.22	− 0.04	− 0.69
HCV1	0.46	− 0.53	− 0.55	0.63	0.56	0.26	*2.91*	2.80	2.59	0.79	− 0.30	0.94	0.44	− 0.05	− 0.43	0.60	− 0.37
HCV2	0.47	− 0.60	− 0.72	0.66	0.31	0.09	2.16	*2.85*	1.84	0.76	− 0.53	0.65	− 0.10	− 0.37	− 0.05	0.50	− 0.32
HCV3	0.39	− 0.73	− 0.77	0.06	0.06	− 0.07	2.63	2.53	*2.32*	0.34	− 0.28	0.92	0.02	− 0.51	− 0.68	0.28	− 0.46
HEV	0.41	− 0.62	− 0.63	0.23	0.35	1.06	− 0.14	− 0.24	− 0.45	*1.89*	− 0.31	− 0.17	0.10	− 0.04	− 0.51	0.54	− 0.39
HIV1	0.96	0.55	0.08	0.77	0.56	0.01	0.05	− 0.06	− 0.27	1.07	*1.89*	0.46	− 0.22	0.06	0.13	0.92	0.23
HIV2	− 0.13	− 0.90	− 0.94	0.02	− 0.29	− 0.07	− 0.09	− 0.20	− 0.41	0.23	0.91	*1.68*	− 0.07	− 0.44	− 0.75	0.09	− 0.66
HTLVI	− 0.57	− 1.29	− 1.43	− 0.35	− 0.55	− 0.51	− 0.06	− 0.17	− 0.38	− 0.21	− 1.08	− 0.87	*2.67*	0.88	− 1.47	− 0.15	− 0.99
HTLVII	− 0.21	− 1.11	− 1.19	− 0.45	− 0.40	− 0.21	0.19	0.08	− 0.13	− 0.09	− 0.92	− 0.59	0.38	*3.30*	− 1.13	− 0.10	− 0.86
WNV	0.10	− 0.50	− 0.70	0.21	− 0.34	− 0.33	− 0.19	− 0.29	− 0.51	0.25	− 0.62	− 0.52	− 0.40	− 0.64	*2.24*	0.00	− 0.82
ZKV	− 0.29	− 0.64	− 0.92	− 0.49	− 0.41	− 0.45	0.12	0.26	0.32	0.68	− 0.96	− 0.68	− 0.52	− 0.78	0.16	*2.07*	− 0.83

Positive results are reported in italics

CK, Chikungunya virus; DEN, dengue; HAV, hapatitis A virus; HCV, hepatitis C virus; HEV, hepatitis E virus; HIV, human immunodeficiency virus; HTLV, human T-cell lymphotropic virus; WNV, West Nile Virus; ZKV, Zika Virus; NC, negative control

**Table 3:** Multi-pathogens-mix test results based on log ratio

	MPM1	MPM2	MPM3	MPM4
CK	*3.42*	*3.37*	0.24	*2.25*
DEN1	*3.14*	*3.10*	*1.80*	*2.45*
DEN2	1.11	1.10	− 0.23	0.35
DEN3	*2.72*	1.19	*3.00*	0.51
DEN4	1.31	1.19	0.83	0.59
HAV	*1.33*	0.12	*2.18*	− 1.13
HCV-1a	*2.53*	0.61	*2.72*	− 0.59
HCV-2a	*2.16*	0.66	*2.44*	− 0.65
HCV-3	*2.45*	0.65	*2.60*	− 0.72
HEV	*1.64*	0.67	*1.21*	− 0.71
HIV-1	1.05	1.24	1.11	− 0.36
HIV-2	0.13	0.24	0.17	− 0.81
HTLV-I	− 0.17	0.06	− 0.07	− 1.37
HTLV-II	− 0.02	0.12	− 0.09	− 1.07
WNV	*1.63*	*1.65*	0.11	− 0.03
ZKV	*3.09*	*3.04*	0.30	*1.98*

Positive results are reported in italics

MPM1, CK, DEN1, DEN3, HAV, HCV-1a, HEV, WNV, ZKV; MPM2, CK, DEN1, WNV, ZKV; MPM3, DEN3, HAV, HCV-1a, HEV; MPM4, CK, DEN1, ZKV

### Individual and mixed viral detection

CHIKV, DENV1–4, HAV, HCV genotypes 1a, 2b, and 3, HIV-1, 2, WNV and ZIKV had 10^2^ copies/mL limits of detection. The lowest detectable level for HEV was 10^4^ copies/mL (Table 4). The analytical sensitivity for each assay was determined using a concentration range based on the clinical requirement for pathogen detection. There were no false negatives or false positives when testing the positive plasma. In the presence of very low pathogen concentrations, the log ratio was at the borderline level so the results were qualified according to double level analysis (at least 50% of the probes generated a fluorescence signal above the set threshold). In the presence of negative plasma samples, the log ratio value was always negative (Table 2).

**Table 4:** Pathogen chip performance based plasma panel test results

Pathogen	Copies/mL	pos/total	qPCR validation
Chikungunya	10^3^	1/1	Y
Chikungunya	10^2^	4/4	Y
Dengue1	10^3^	3/3	Y
Dengue1	10^2^	2/2	Y
Dengue1	10^1^	0/1	Y
Dengue2	10^3^	3/3	Y
Dengue2	10^2^	3/3	Y
Dengue2	10^1^	0/1	Y
Dengue3	10^3^	3/3	Y
Dengue3	10^2^	3/3	Y
Dengue3	10^1^	0/1	Y
Dengue4	10^3^	3/3	Y
Dengue4	10^2^	3/3	Y
Dengue4	10^1^	0/1	Y
HAV	10^3^	2/2	Y
HAV	10^2^	2/2	Y
HCV-1a	10^3^	3/3	Y
HCV-1a	10^2^	3/3	Y
HCV-2a	10^2^	2/2	Y
HCV-3	10^2^	2/2	Y
HEV	10^4^	3/3	Y
HEV	10^3^	0/2	Y
HEV	10^2^	0/2	NA
HIV-1	10^3^	2/2	y
HIV-1	10^2^	2/2	y
HIV-2	10^3^	3/3	y
HIV-2	10^2^	3/3	y
HTLV-I	10^3^	2/2	y
HTLV-I	10^2^	2/2	y
HTLV-II	10^3^	2/2	y
HTLV-II	10^2^	2/2	y
WNV (NY99)	10^5^	1/1	y
WNV (NY99)	10^4^	1/1	y
WNV (NY99)	10^3^	3/3	y
WNV (NY99)	10^2^	4/4	y
WNV (NY99)	10^1^	0/2	NA
ZIKV PRVABC60	10^3^	3/3	Y
ZIKV PRVABC61	10^2^	3/3	Y
ZIKV PRVABC62	10^1^	0/2	Y
ZIKV FSS13025	10^3^	3/3	Y
ZIKV FSS13025	10^2^	3/3	Y
ZIKV FSS13025	10^1^	0/2	Y
MPM1	10^5^–10^3^	3/3	y
MPM2	10^5^–10^3^	3/3	y
MPM3	10^5^–10^3^	3/3	y
MPM4	10^5^–10^3^	3/3	y

NA, not applicable

A mix of different positive plasma samples at different concentrations was simultaneously tested in a single experiment. Four different combinations were generated. The multi-pathogens-mixes were composed of 8 (CHIKV, DENV1, DENV3, HAV, HCV1a, HEV, WNV and ZIKV), 4 (CHIKV, DENV1, WNV and ZIKV), 4 (DENV3, HAV, HCV1a and HEV) and 3 (CHIKV, DENV1 and ZIKV) different pathogens, respectively at a concentration range from 10^5^ to 10^3^ copies/mL (Tables 3, 4).

Among the 99 positive samples tested at a concentration ranging from 10^5^ to 10^2^, 92 out of 92 samples were correctly detected (excluding HEV). HEV was detected by microarray in 3 of 7 qPCR positive samples (42%) at a final concentration of 10^4^ copies/mL. No specific signal was detected below this value. Overall, there were 21 qPCR-positive samples that were not detected in microarray format because the concentration was below the limit of detection of the platform (< 10^2^). All pathogens were detected without interference among the targets in all 4 mixed combinations (Table 3).

All the samples tested (single or multiple pathogen mixtures) were replicated at least 3 times with at least a 1 week interval between the experiments, in order to test the reproducibility of the results. The consistency of positive results across the different arrays confirmed that the array design combined with the double level analysis model, performed well.

### RT-qPCR confirmation

All positive results were confirmed and the copy numbers for each pathogen were calculated to define the limit of the detection for each species on the array (Additional file 4: Table S2).

## Discussion

Blood donors in the U.S. are routinely screened for ten infectious agents; six using nucleic acid detection methodology and four with immunoassays. However, more than thirty pathogens pose a significant threat to the blood supply [3, 6]. Multiplex screening exists only for detection of HBV, HCV and HIV. Recent emergent threats, such as WNV and ZIKV, have been added to the growing list of individual tests required for blood donor screening.

Microarrays are miniaturized detection platforms consisting of short (25-mer to 70-mer) single-stranded oligonucleotide probes deposited onto a solid substrate. A microarray-based platform has the advantage of having a small solid surface upon which thousands of oligonucleotide probes can be printed to simultaneously detect a multiplicity of target pathogens [16–18]. Fluorescently labeled nucleic acid samples are hybridized to the microarray, and hybridization patterns are analyzed to identify the specific pathogens that are present. A dedicated multi-pathogen detection platform for blood donor screening with a short processing time and well-defined processes for data analysis could significantly improve the safety of the blood supply.

We described here early-stage design, development and validation of a microarray-based pathogen chip to screen blood donors for transfusion–transmitted RNA viruses. We developed an array design, sample material preparation method, hybridization conditions and a single (non-primer specific) amplification step for detection of 16 viral pathogens without compromising performance. We determined the limit of detection of these viruses in human plasma specimens tested individually, or as a mixture of up to eight different pathogens. We also developed a method for data analysis and interpretation capable of discriminating multiple viruses in the same sample set.

The design included multiple gene targets for each pathogen genome in order to increase the sensitivity of the platform. The intensities of individual probes in a probe set can vary substantially and the contributions of peak intensities within the set can be obscured through averaging, so if the virus species are under-represented by the array probe sets, the detection of that specific target can be missed. In order to increase the platform coverage of the virus landscape to which the array is designed to target, the design strategy was to balance the number of probes for each pathogen to a final count of 90–110 probes previously determined to be the most reactive. Another fundamental step to increase the level of sensitivity was to increase the quantity of RNA targets for hybridization through sample amplification. We designed a novel workflow combining two different applications, in use independently, but never combined, to increase the concentration of target nucleic acids. We applied a sequence independent, single primer linear isothermal amplification (Ribo-SPIA) procedure for preparation of target cDNA [19]. This approach allowed detection of low viral RNA copy numbers without the necessity of designing specific primers for each pathogen. Nearly all known PCR-positive samples were detected on the platform and all the probes generated a strong signal specific for each positive plasma specimen analyzed. There were no specific signals produced by negative control plasma, and only in a few arrays were random nonspecific intensity signals produced. This indicated that the generation of cDNA instead of amplified RNA, followed by Cy3 labeling and hybridization based on a DNA application, worked almost perfectly. Among the 99 positive samples tested at a concentration ranging from 10^5^ to 10^2^ copies/mL, 95 (96%) were correctly detected at all concentrations. Only HEV failed to be detected in 4 of 7 PCR-positive samples with a 10^4^ copies/mL limit of detection.

Blood donors may uncommonly be co-infected with more than one agent, and therefore multiple pathogens need to be distinguished simultaneously. The mixes of between 3 and 8 different pathogens at different concentrations were identified, with no interference among the targets, at a 10^3^ limit of detection.

The potential of some viruses to mutate with high frequency, and the need to rapidly develop a new test for emerging pathogens in response to outbreaks, are important factors to consider during multi-pathogen detection platform design. An ideal multiplex device should have the flexibility to expand to detect emerging agents and, at the same time, have a relatively short validation time frame. We were able to modify the probe design, and manufacture an updated version of the pathogen chip in less than 4 weeks during the 2016 ZIKV outbreak. The expansion with ZIKV-specific probes did not alter platform performance.

The aims of the study were to optimize platform performance, improve the limits of detection and develop a method for data analysis and interpretation. The use of spiked specimens was useful to evaluate the reproducibility and practicality of the new platform. The spiked specimens represented a standardized method to facilitate the evaluation of probe design, amplification strategies, and bioinformatics analysis among different experiments. The next phase of development will include attainment specimens from patients who had been infected with one or more agents and evaluation of platform performance against a blinded panel of known positive and negative clinical samples.

Overall, this microarray-based pathogen-chip met the predicted discriminating ability for multi-pathogen detection. Using optimized methods for sample preparation, labelling, and hybridization, this platform achieved simultaneous molecular detection of sixteen transfusion–transmitted RNA viruses. In this format, oligonucleotide probes, rigorously validated for specificity, sensitivity, and reproducibility, showed strong correlation with independent RT-qPCR quantitation, down to a level of 10^2^ copies/mL. These attributes, combined with successful simultaneous discrimination between up to eight pathogens and the potential for rapid addition of new detection targets, make this design a promising step towards improvements in donor blood safety. An integrated pathogen chip to detect RNA and DNA viruses, bacteria and parasites is in development.

## Conclusion

In this proof of concept study, we evaluated the performance of a customized microarray-based pathogen chip for simultaneous detection of 16 RNA viruses in human plasma samples that is designed to have the flexibility to expand to detect emerging agents in a relatively short time frame. Following proof of concept validation, the platform could be commercially developed for blood donor screening and diagnostic use with potential for other blood pathogen screening applications. The ability to rapidly test both large numbers of donors or a single donor sample (with a consistent cost per sample) for all pathogens of concern with a single multiplex test, keeping the same sensitivity and specificity of single tests would significantly enhance the safety of the blood supply.

## Additional files

**Additional file 1: Table S1.** Viral pathogens used in testing the Pathogen Chip.

**Additional file 2: Figure S1.** Example of probes intensity distribution for positive detection. A) Dengue Virus 1 RNA (10^3^ copies/mL) was hybridized to the Pathogen Chip containing 101 probes covering the reference genome. The RNA was extracted from Dengue 1 positive plasma sample. Red numerals indicate the genomic region covered by the specific probes; 1, RNA-dependent RNA polymerase NS; 2, anchored capsid protein C; 3, envelope protein E; 4, membrane glycoprotein precursor; 5, nonstructural protein NS1; 6, nonstructural protein NS2A; 7, nonstructural protein NS2B; 8, nonstructural protein NS3; 9, nonstructural protein NS4A; 10, nonstructural protein NS4B. B) Four samples positive for Dengue 1 Viruses assayed on pathogen chip at different concentration. Probes generating more intense signal and producing higher percentage coverage of the specific genome across the different experiments were selected in the final design.

**Additional file 3: Figure S2.** Amplification method and Pathogen Chip assay performance assessed using positive control Viral RNAs. A) SPIA amplification vs standard (STD) method. cDNA concentration after amplification for four representative viral RNAs. Starting RNA concentration was < 10 ng/μL each. SPIA method showed better amplification. B) Pathogen chip assay performance 1. Orange bars are the mean of Cy3 signal for the Chikungunya and West Nile probes hybridized to test samples positive for CHIKV and WNV and a negative plasma sample. Only probes specific to target showed a specific hybridization signal. No signal for negative plasma. C) Pathogen chip assay performance 2. Detection responses of four representative samples (Dengue 4) were measured over a dilution series from 10,000 to 10 genomic copies per sample. Orange bars are the mean of Cy3 signals for all probes to the indicated viruses hybridized to test samples.

**Additional file 4: Table S2.** Validation of pathogen chip detecion results.

## Data Availability

The datasets used and/or analyzed during the current study are available from the corresponding author upon reasonable request. This microarray-based pathogen chip is protected under provisional patent number U.S. Patent Application No. 62/799,482.
