# Supplementary figures and images for: A microarray-based pathogen chip for simultaneous molecular detection of transfusion–transmitted infectious agents
Source: J Transl Med. 2019 May 14;17:156. doi: 10.1186/s12967-019-1905-4 (PMC6518760; doi:10.1186/s12967-019-1905-4)

Dengue 1 probes distribution

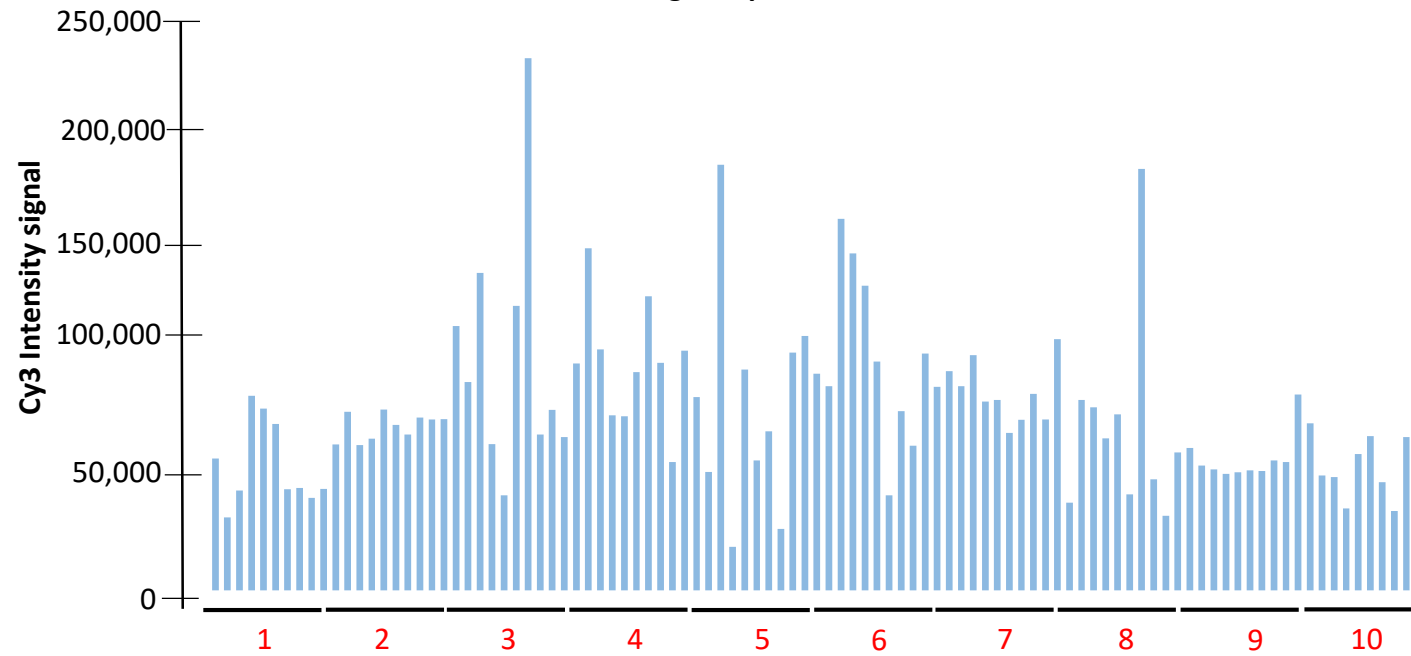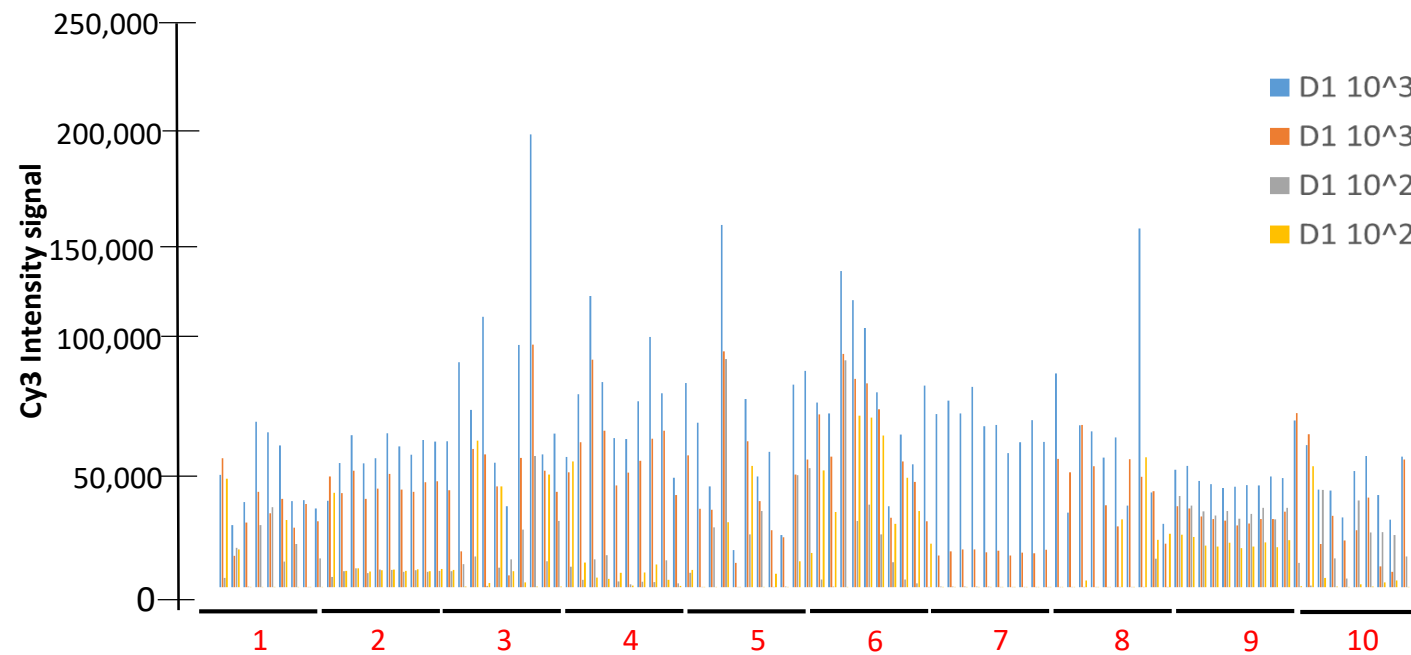

Supplement: Supplementary file 2 — Additional file 2: Figure S1. Example of probes intensity distribution for positive detection. A) Dengue Virus 1 RNA (103 copies/mL) was hybridized to the Pathogen Chip containing 101 probes covering the reference genome. The RNA was extracted from Dengue 1 positive plasma sample. Red numerals indicate the genomic region covered by the specific probes; 1, RNA-dependent RNA polymerase NS; 2, anchored capsid protein C; 3, envelope protein E; 4, membrane glycoprotein precursor; 5, nonstructural protein NS1; 6, nonstructural protein NS2A; 7, nonstructural protein NS2B; 8, nonstructural protein NS3; 9, nonstructural protein NS4A; 10, nonstructural protein NS4B. B) Four samples positive for Dengue 1 Viruses assayed on pathogen chip at different concentration. Probes generating more intense signal and producing higher percentage coverage of the specific genome across the different experiments were selected in the final design. [file 12967_2019_1905_MOESM2_ESM.pdf]

### cDNA Yields

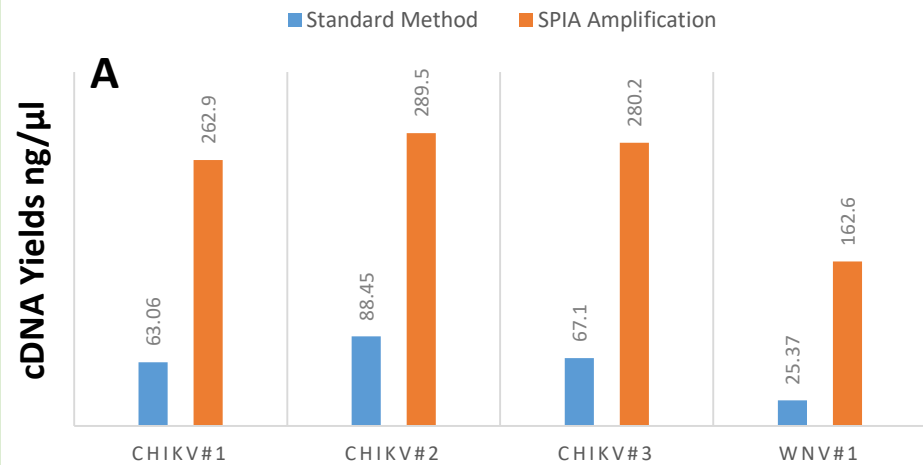

### Pathogen chip assay performance 1

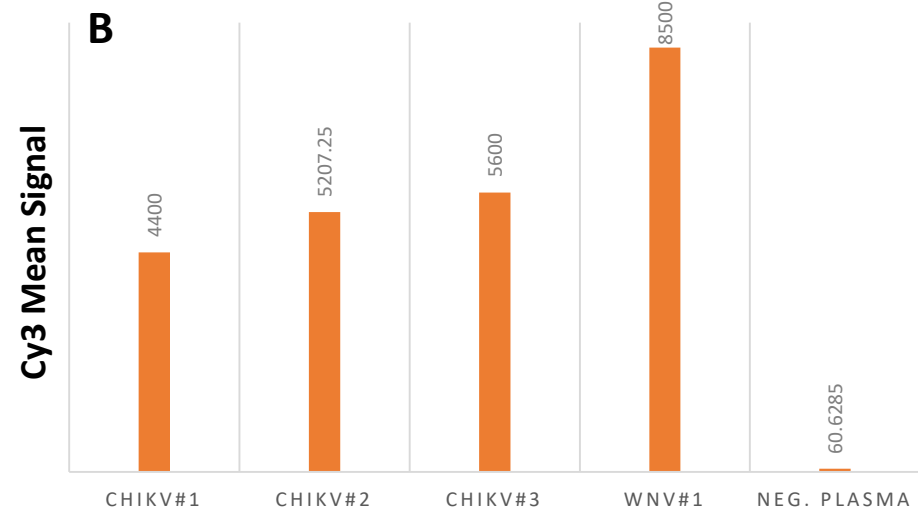

### Pathogen chip assay performance 2

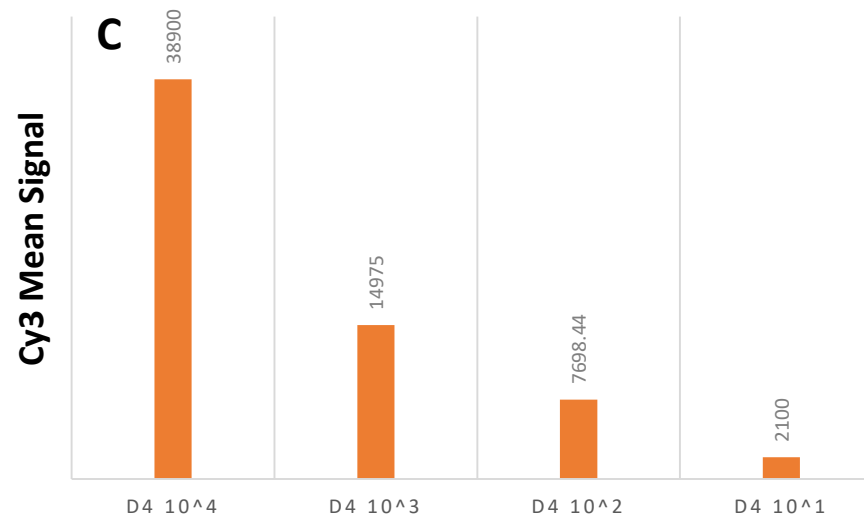

Supplement: Supplementary file 3 — Additional file 3: Figure S2. Amplification method and Pathogen Chip assay performance assessed using positive control Viral RNAs. A) SPIA amplification vs standard (STD) method. cDNA concentration after amplification for four representative viral RNAs. Starting RNA concentration was < 10 ng/μL each. SPIA method showed better amplification. B) Pathogen chip assay performance 1. Orange bars are the mean of Cy3 signal for the Chikungunya and West Nile probes hybridized to test samples positive for CHIKV and WNV and a negative plasma sample. Only probes specific to target showed a specific hybridization signal. No signal for negative plasma. C) Pathogen chip assay performance 2. Detection responses of four representative samples (Dengue 4) were measured over a dilution series from 10,000 to 10 genomic copies per sample. Orange bars are the mean of Cy3 signals for all probes to the indicated viruses hybridized to test samples. [file 12967_2019_1905_MOESM3_ESM.pdf]
